# Light and Shadow of Na-Glucose Cotransporter 2 Inhibitors in the Treatment of Diabetes Mellitus: Points for Improvement Based on Our Clinical Experience

**DOI:** 10.1155/2024/3937927

**Published:** 2024-01-25

**Authors:** Akihiro Sonoda, Toshio Shimada, Kohei Saito, Rieko Kosugi, Yoshitaka Taguchi, Tatsuhide Inoue

**Affiliations:** ^1^Clinical Research Center, Shizuoka General Hospital, Shizuoka, Japan; ^2^Department of Clinical Laboratory, Shizuoka General Hospital, Shizuoka, Japan; ^3^Division of Diabetes, Endocrinology, and Metabolism, Shizuoka General Hospital, Shizuoka, Japan

## Abstract

We analyzed the effect of Na-glucose cotransporter 2 inhibitors (SGLT2-I) in diabetic patients visiting our hospital. The study included 236 patients treated with SGLT2-I alone or with codiabetic drugs for at least two years. We analyzed overtime changes in glycosylated hemoglobin A1c (HbA1c) in the patients by repeated analyses of variance (ANOVA) and evaluated the therapeutic effect. HbA1c levels decreased significantly in the first six months after treatment. Afterward, they leveled off and increased slightly over the next two years. Six months after treatment, the mean (SD) of HbA1c was 8.19 (1.46) %; the mean difference dropped by 0.91%, and HbA1c in mild DM2 did not drop by below 8.0%. Overall, there was only a slight improvement. We performed multivariate logistic regression analysis using a model with or without improvement as the objective variable and several explanatory variables. Na and Hct were significant factors. They increased considerably over six months and then leveled off. eGFR significantly reduced in the hyperfiltration group six months after treatment. The annual decline rate in eGFR was also faster, even in the nonhyperfiltration group than in the healthy subjects, which may be a characteristic of renal clearance in SGLT2-I treatment. In conclusion, SGLT2-I is an excellent antidiabetic, nephroprotective agent to eliminate hyperfiltration, but unfortunately, SGLT2-I alone does not have enough power to reduce blood glucose levels. SGLT2-I, with insulin or insulin secretagogues, enhances insulin resistance, induces hyperinsulinemia, and exacerbates type 2 DM. In contrast, SGLT2-I, with noninsulin antidiabetic agents and a low-carbohydrate diet, may bring better results.

## 1. Introduction

About ten years have passed since Na-glucose cotransporter 2 inhibitors (SGLT2-I) were launched as a new treatment for type 2 diabetes mellitus (type 2 DM). Before launching SGLT2-I, first-line drugs for treating hyperglycemia have been insulin secretagogues, insulin sensitizers, gluconeogenesis inhibitors, and glucose absorption inhibitors, either alone or in combination. The mechanism of action of SGLT2-I is to inhibit glucose reabsorption in the proximal tubules of the kidneys and increase its excretion into the urine. SGLT2-I, in combination with biguanides (BGs), is expected to be more effective in lowering hyperglycemia [1].

Initially, the use of SGLT2-I began by offering insufficient scientific information. Safety was a concern, lacking detailed information on normoglycemic ketoacidosis and genitourinary infections. There were also concerns about increased cerebrovascular events due to fluid volume reduction caused by diuretic effects [2, 3]. Accumulated clinical experience, conference presentations, and publications on SGLT2-I dispelled these concerns. On the other hand, the diuretic action of SGLT2-I brought back various metabolic improvements [4, 5].

The hypoglycemic action of SGLT2-I by excreting glucose in the urine may make a decisive difference in treating type 2 DM because, unlike insulin secretagogues and other hypoglycemic drugs that stimulate insulin secretion, SGLT2-I does not induce hyperinsulinemia [6]. SGLT2-I is a unique antidiabetic agent suppressing systemic damage from type 2 DM by mitigating and avoiding glucotoxicity [7–9].

Nevertheless, type 2 DM patients have not been well treated for over half a century. We investigated the effect of SGLT2-I alone or in combination on type 2 DM patients and have made an effort to resolve the issues with the type 2 DM treatment and to search for new reinforcement therapy.

We must escape from a maze as soon as possible to reach the type 2 DM therapy target.

### 1.1. Objectives of the Study

Since introducing insulin to type 2 DM therapy, patients with type 2 DM have not undergone enough treatment for over half a century. Ten years have almost passed since launching antidiabetic drugs with a new concept that can lower blood glucose levels without increasing blood insulin levels. To use SGLT2-I for urgent escape from the therapeutic maze and open a new door to type 2 DM treatment, we conducted this study based on real-world data, exploring the merits and demerits of the drug.

### 1.2. Subjects and Methods

In clinical practice, we retrospectively studied the efficacy of SGLT2-I on Type 2 DM patients who visited our clinic after launching SGLT2-I in May 2014 through January 2020. Of the 349 patients diagnosed with type 2 DM and treated with SGLT2-I during the study period, we enrolled 236 patients (152 men and 84 women) who continued to receive SGLT2-I alone or along with other antidiabetic drugs during the 2-year study period.

## 2. Methods of Data Analysis

### 2.1. Trends in HbA1c after Administering SGLT2-I

We analyzed HbA1c levels at starting, six months, one year, 1.5 years, and two years after treatment, using a one-way repeated analysis of variance. We performed multiple comparisons (intragroup comparison) with Bonferroni correction.

We calculated the difference as [(HbA1c at each post-treatment time point) − (HbA1c level before treatment)]% for tracing. For each patient, in addition, we calculated the change rate in HbA1c from the following equation: [(HbA1c at each post-treatment time point) − (HbA1c before treatment)]%/(HbA1c before treatment)% × 100. We classified patients by the median of change rate (MCR) in HbA1c six months after treatment (−8.4%) into two groups: an improvement group (MCR ≤ −8.4%) and a nonimprovement group (−8.4% < MCR). We analyzed time series data of HbA1c for two years by two-way repeated analysis of variance (interaction: group × time). To offset differences in HbA1c (%) at starting treatment, we divided the HbA1c (%) difference as follows: [(post-treatment HbA1c (%) − pretreatment HbA1c (%)] with individual matched pretreatment HbA1c (%) to calculate the standardized change rate by multiplying 100. If the main effect and interaction were significant, we determined the primary outcome and performed multiple comparisons with Bonferroni correction. If the interaction was not significant, we conducted multiple comparisons only for the primary outcome.

As HbA1c increased slightly in the second year after treatment, we classified the patients into two groups based on the median difference in HbA1c between 6 months and two years after treatment. We defined the group with an increment in the difference of HbA1c ≥1.0% as the worsening group and the group with a decrement in the difference of HbA1c ≤−1.0% as the improving group.

We analyzed the change rate in HbA1c using a two-way repeated analysis of variance.

### 2.2. Analysis of Factors Influencing Improvement in Diabetes

We made a multivariate logistic regression model to analyze influencing factors.

In the same way as the procedure described above, we classified patients by the median change rate (MCR = −8.4%) in HbA1c at six months after treatment into two groups: one group (MCR ≤ −8.4%) and the other group (MCR > −8.4%) at six months after treatment as the objective variable.

We adopted ALT (alanine aminotransferase), *γ*-GTP (*γ*-glutamyl-transpeptidase), TG (triglycerides), LDL-C (low-density lipoprotein-cholesterol), HDL-C (high-density lipoprotein-cholesterol), Na (sodium), K (potassium), UA (uric acid), CRE (creatinine), BUN/CRE (urea nitrogen creatinine ratio), and Hct (hematocrit) as the explanatory variables.

Multivariate logistic regression analysis extracted Na and Hct as affecting type 2 DM improvement factors.

We used one-way and two-way repeated analyses of variance for both variables. Based on the results, we hypothesized that the effect of SGLT2-I was closely related to dehydration through renal glomerular filtration rate, and further analysis focused on eGFR.

Since the eGFR at starting treatment differed individually, we standardized eGFR as the change rate from the following equation: [(eGFR at each time point after treatment) − (eGFR before treatment)]/(eGFR before treatment) × 100.

We performed a two-way repeated analysis of variance using eGFR from starting treatment to 2 years. However, we excluded sex and age from adjusting because the eGFR calculation formula had already adjusted sex and age.

eGFR declines with age, so we defined the age-specific reference values based on eGFR in the general Japanese population from 10-year longitudinal follow-up data [10]. We calculated hyperfiltration as an eGFR value beyond the upper limit of 2 SD [11]. Using the age-specific definition, we divided the patients into two groups. One group was a hyperfiltration group that exceeded the age-specific reference value in the eGFR. The other group was nonhyperfiltration, which did not exceed the age-specific reference value in the glomerular filtration rate.

### 2.3. Statistical Analysis Software Used for the Analysis

We performed one-way, two-way repeated variance, and multivariate logistic regression analyses using “R, EZR, js-STAR version 9.0.0 j.” A *p* value <0.05 was considered statistically significant.

### 2.4. Ethical Considerations

The protocol for this study was reviewed and approved by the Clinical Research Ethics Committee of Shizuoka General Hospital (approval number: SGHIRB—number: 2020086).

## 3. Results

### 3.1. Background of the Patients

As shown in Table 1, 236 patients (152 males and 84 females) underwent the same prescription for two years after treatment. The mean age of study patients was 61.4 (25–87) years, and their mean BMI before SGLT2-I treatment was 27.1 kg/m^2^ (18.4–50.8 kg/m^2^).

### 3.2. Trends in HbA1c during the First Two Years of Treatment

**Figure 1:**
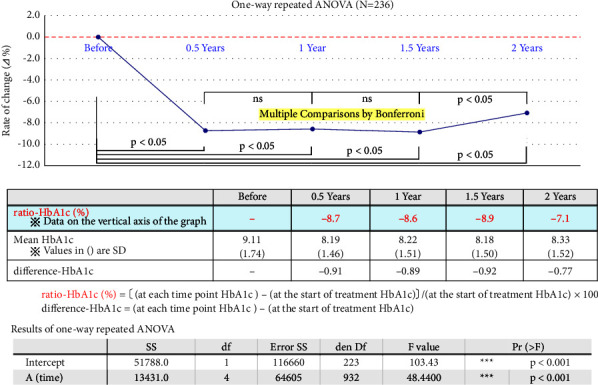
Rate of change in HbA1c over two years after initiation of treatment by one-way repeated ANOVA. HbA1c at the start of treatment, six months, one year, 1.5 years, and two years later were analyzed by one-way repeated analysis of variance. We used the Bonferroni correction method to adjust multiple comparisons between points (within-group comparisons): HbA1c decreased significantly (*p* < 0.05) at six months after the start of treatment, then remained almost unchanged but showed an upward trend again at two years. Intercomparisons of HbA1c at six months, one year, 1.5 years, and two years after the start of treatment showed no significant differences.

We analyzed the change rate in HbA1c from the start of treatment by one-way repeated analysis of variance, and Figure 1 shows the results. Six months after beginning treatment, HbA1c levels decreased significantly (*p* < 0.05) and then leveled off but began to increase again two years later (*p* < 0.05).

All patients' mean (SD) of HbA1c was 8.19 (1.46)% six months after treatment and decreased by 0.91 percent from starting treatment. The mean (SD) of HbA1c two years after treatment was 8.33 (1.52)%, which dropped by 0.77% from the beginning of treatment but showed an increasing trend after two years.

**Figure 2:**
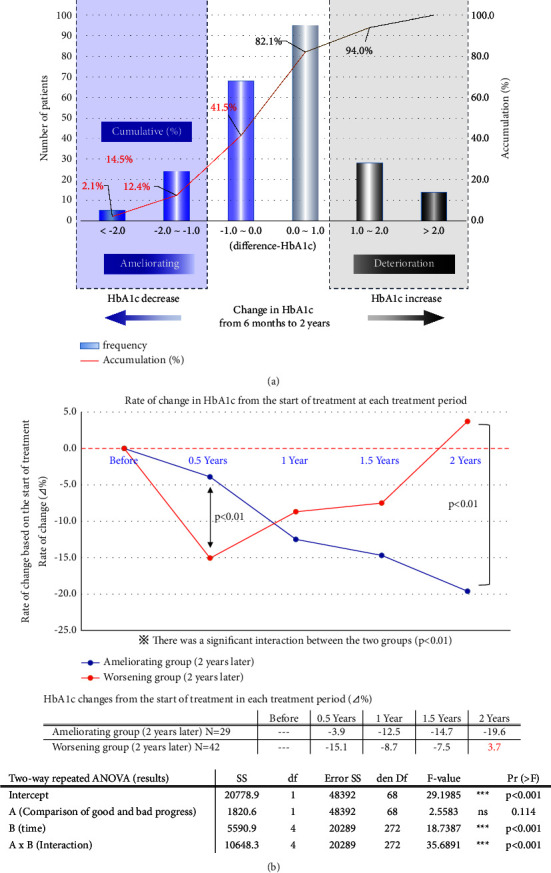
(a) Change in HbA1c from six months to two years. For each of the 236 patients, we calculated the difference between the HbA1c at six months and two years after the start of treatment, and the histogram showed the mean difference (difference-HbA1c). 41.5% of the patients tended to improve, and 58.5% tended to worsen. Only 14.5% of the patients improved by 1.0% or more. (b) Rate of change (⊿%) in HbA1c between the groups whose HbA1c levels ameliorated and worsened from six months to two years after the start of treatment (two-way repeated analysis of variance). Ameliorating group: patients whose HbA1c level decreased by 1.0% or more. Worsening group: patients whose HbA1c level increased by 1.0% or more.

We calculated the difference in HbA1c between 6 months and two years after treatment, and Figure 2(a) shows the frequency distribution of difference inHbA1c. 41.5% of patients showed an improving trend, and 58.5% showed a slightly worsening trend.

When we observed the overtime change in HbA1c between the improving and worsening groups, the improving group showed a constant decrease in HbA1c throughout the study period. In contrast, the worsening group showed a dramatic decrease in HbA1c at the start of the follow-up and a marked increase after two years (Figure 2(b)).

**Figure 3:**
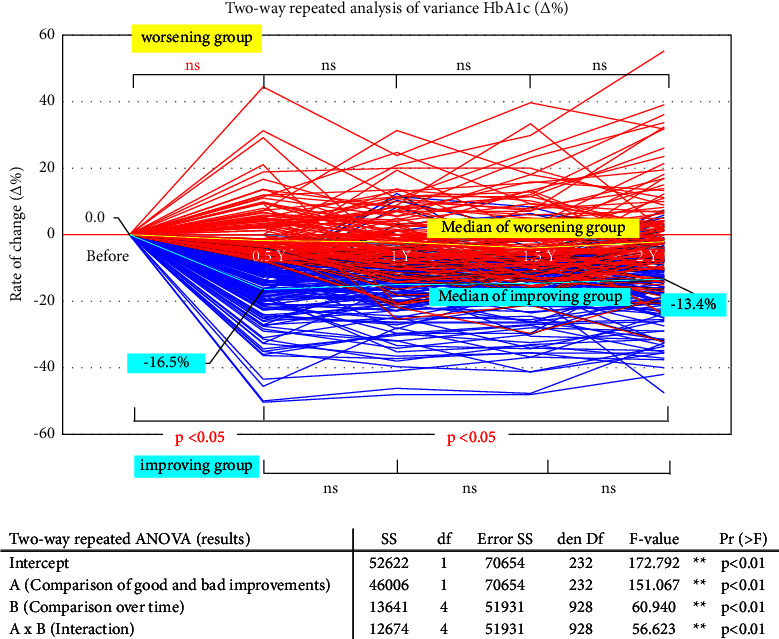
Comparison of changes in HbA1c over time in two groups with and without improvement in diabetes mellitus. The mean rate of change in HbA1c in the improving group (bold blue line) decreased significantly in the first six months of treatment (*p* < 0.05), remained almost unchanged after that, and showed an upward trend again after two years. In the nonimproving group (bold yellow line), there was little change (*p* > 0.10) throughout the two-year follow-up period from the start of treatment (Bonferroni correction method for multiple comparisons within groups).

**Figure 4:**
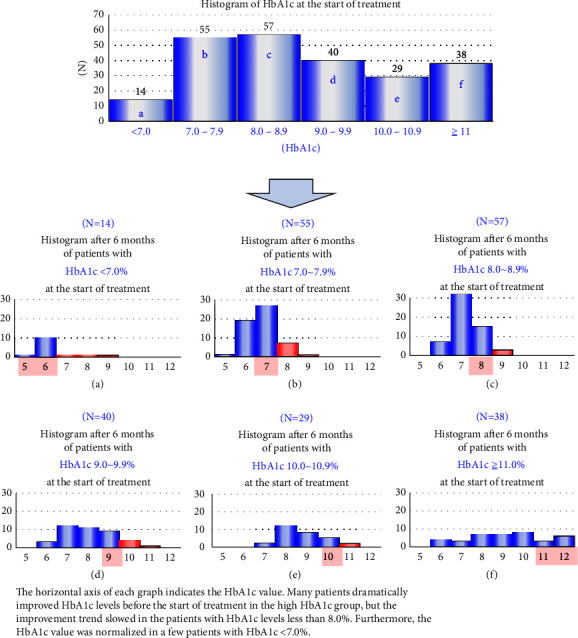
HbA1c histogram at the beginning of treatment (upper middle graph) and after six months (graphs (a)–(f)). Columns from (a) to (f) in the upper figure show the histogram of HbA1c at the start of treatment. The histograms six months after treatment were redrawn below. (a) to (f) histograms show the detailed changes in HbA1c.

We calculated the trends in ratio, HbA1c (%), in the improving and worsening groups. As shown by the bold blue line in Figure 3, the change in mean ratio, HbA1c (%), in the improving group decreased significantly (*p* < 0.05) six months after treatment. After that, it remained flat and showed an increasing trend after two years (*p* < 0.05). The mean specific HbA1c (%) in the worsening group remained unchanged throughout the 2-year follow-up after starting treatment (bold yellow line). Furthermore, multiple comparisons with Bonferroni correction showed no significant differences (*p*  >  0.10). To examine the relationship between diabetes severity and SGLT2-I treatment effect, we subdivided pretreatment HbA1c values into every 1.0% width. The histogram in Figure 4 shows the HbA1c values six months after treatment in each patient group. In the high HbA1c groups (d, e, and f in Figure 4), the improvement in HbA1c significantly and slightly decreased. In particular, the mild diabetic group with HbA1c <8.0% (a and b in Figure 4) showed a slight decrease of about 1%, but none normalized.

### 3.3. Analysis of Factors Influencing Type 2 DM Improvement

Using a multivariate logistic regression model with the presence or absence of type 2 DM improvement as the objective variable, Na (OR = 0.874, 95% CI [0.781, 0.978], *p*=0.019) and Hct (OR = 1.080, 95% CI [1.000, 1.160], *p*=0.042) were extracted as factors significantly related to type 2 DM improvement.

One-way repeated variance analysis of the 2-year changes in these variables showed that both Na and Hct increased significantly at six months and then leveled off; considering the mechanism of action of SGLT2-I, this result may be related to dehydration, closely linked to renal glomerular filtration. Therefore, we focused our analysis on eGFR.

**Figure 5:**
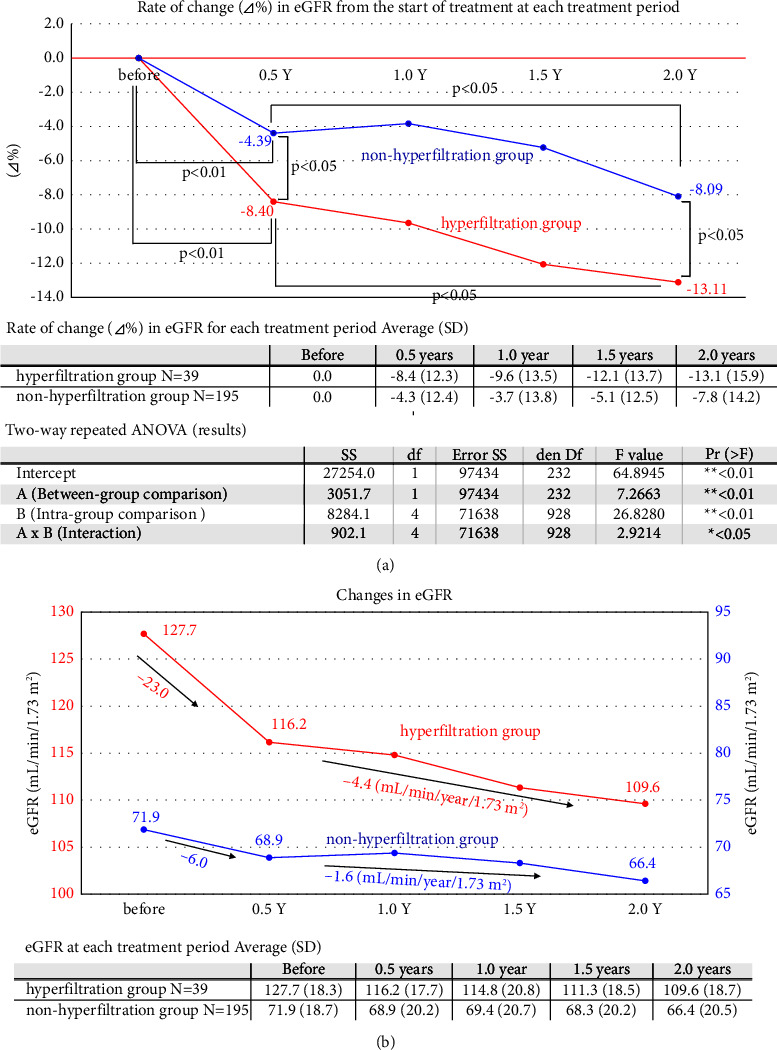
(a) Comparison of eGFR levels over two years from the start of treatment (two-way repeated ANOVA). We performed a two-way repeated analysis of variance for 236 patients subdividing into the hyperfiltration and nonhyperfiltration groups with eGFR. The eGFR in the hyperfiltration group decreased significantly during the first six months of treatment (*p* < 0.01) and then slowly declined during the follow-up period. In the nonhyperfiltration group, eGFR also decreased significantly (*p* < 0.01) during the first six months of treatment and then slowly reduced during the follow-up period. There was a significant difference between the two groups (*p* < 0.01), and there was an interaction in the pattern of variation (*p* < 0.05). (b) Changes in eGFR between hyperfiltration group and nonhyperfiltration group. Figure 5(b) shows the mean eGFR in the line graphs over the two-year follow-up period in the hyperfiltration group and the nonhyperfiltration group. The hyperfiltration group showed a rapid decrease in eGFR of −23.0 mL/min/year/1.73 m^2^ in terms of the annual rate of decline from the start of treatment to six months after the start of treatment and the annual rate of decrease from then to two years after the start of therapy was −4.4 mL/min/year/1.73 m^2^. The rate of decline was slightly slower but still fast. The nonhyperfiltration group also showed a rapid decrease (−6.0) until six months after the start of treatment and then a slow decline (−1.6).

Based on eGFR, we divided patients into the hyperfiltration and nonhyperfiltration groups.  Figure 5(a), analyzed by a two-way repeated analysis of variance, showed an interaction between the two groups over two years (*p* < 0.05). In the hyperfiltration group, there was a markedly decreased eGFR in the first six months of treatment (*p* < 0.01), followed by a gradual decline. In the nonhyperfiltration group, eGFR decreased slowly (*p* < 0.01) during the first six months of treatment, and the subsequent decrease was also gradual and sustained.

We show the mean eGFR of the hyperfiltration and nonhyperfiltration groups during the 2-year follow-up period. The hyperfiltration group showed a rapid decrease in eGFR of −23.0 mL/min/year/1.73 m^2^ until six months after the start of treatment, followed by a decline of −4.4 mL/min/year/1.73 m^2^ until two years after treatment. After that, the rate of decline slowed but still had a relatively rapid decline rate. The nonhyperfiltration group also declined rapidly to −6.0 mL/min/year/1.73 m^2^ until six months after starting treatment and afterward slowly lowered to −1.6 mL/min/year/1.73 m^2^ (Figure 5(b)).

## 4. Discussion

Type 1 DM has seen a dramatic improvement in treatment since the discovery of insulin by Banting et al. Insulin therapy has saved many patients with type 1 DM. In contrast, type 2 DM has remained among the most intractable diseases for over half a century. Various antidiabetic drugs, including insulin and insulin secretagogues, have been primarily used to save their lives. Despite tremendous efforts, we have not reached a breakthrough point yet. Generally, we can understand that type 2 DM results from increased insulin resistance, closely related to poor lifestyle habits, interfering with insulin function. As a result, glucose metabolism becomes impaired due to increased insulin resistance, and type 2 DM progresses secretly or quietly to develop hyperglycemia and hyperinsulinemia.

The European Association for the Study of Diabetes 2009 highlighted the increased cancer risk in patients with long-term insulin glargine use. As we know well, insulin has a cell proliferative action. According to previous papers, recombinant insulin products potentiated this action, leading to an increased risk of carcinogenesis and atherogenesis through downregulating the antiatherogenic and carcinogenic pathways with long-term use [12–16].

Most previous antidiabetic drugs induce insulin directly or indirectly, while SGLT2-I does not cause hyperinsulinemia. SGLT2-I lowers blood glucose levels by inhibiting glucose reabsorption in the proximal tubule and increasing urinary glucose excretion. We must stop insulin-dependent therapy, which has been complacent for over half a century. We need the polypharmacy of insulin-independent antidiabetic therapies that augment and supplement the action of SGLT2-I [17, 18].

We must establish a new therapeutic strategy using SGLT2-I and other antidiabetic drugs without stimulating insulin secretion. Under this concept, we conducted this study to realize the current situation and find a new direction for future clinical practice.

Insulin and insulin secretagogues primarily treated our patients. We must seriously reflect on the fact that the treatment of type 2 DM has been heavily dependent on insulin and insulin secretagogues.

### 4.1. Treatment-Induced Changes in HbA1c during the First Two Years

As an indicator of the effect of SGLT2-I, we evaluated outcomes by observing changes in HbA1c over time after starting antidiabetic treatment.

HbA1c levels fell sharply in the first six months after treatment, then plateaued, followed by a significant increase, in line with other reports.

A network meta-analysis of RCTs reported a mean decrease in HbA1c of 0.59% to 1.23% with SGLT2-I monotherapy, and our results were consistent with those reports.

To elucidate the cause of the increase in HbA1c in the second half of the observation period, we compared the patients whose HbA1c increased by more than 1.0% with the patients whose HbA1c decreased by more than 1.0% from starting treatment to two years after treatment. In the patients whose HbA1c rapidly declined six months after treatment, HbA1c turned around and rose to above baseline. Such a change in HbA1c is unexpected; as far as we know, no one has reported such a case. We also examined the background of the worsening group but found no significant causes in lifestyle-related factors. Therefore, poor adherence does not apply, and the reasons for most of this group still need to be clarified.

### 4.2. Factors Associated with Improvement of Type 2 DM

Multivariate logistic regression analysis extracted sodium and Hct as significant factors improving type 2 DM. Still, the results were attributed to increased sodium reabsorption via SGLT2 in the proximal tubules and regulation via glomerular feedback mechanisms. When SGLT2-I inhibits sodium reabsorption, the sodium influx into the distal tubule increases. The macula densa sensing the sodium concentration constricts the glomerular influx arterioles, decreasing GFR due to decreasing intraglomerular pressure. This mechanism acts as a nephroprotective effect by relieving the kidneys from overload [19–21]. In SGLT2-I therapy, we are alert to dehydration due to osmotic diuresis associated with glucose loss without adequate fluid supply, and we understand that elevated sodium reflects the effects of dehydration. We also believe that the rise in Hct, like the rise in sodium, generally reflects the effects of dehydration; Sano and Goto reported that SGLT2-I alleviates hypoxia due to overloaded glucose reabsorption in the proximal tubules of type 2 DM, restores reduced erythropoietin-producing capacity, and increases Hct [19]. Hypoxia in the proximal tubules induces fibrosis of erythropoietin-producing fibroblasts, causing them to differentiate into myofibroblasts and reducing their erythropoietin -producing capacity [20]. In patients with type 2 diabetes, serum erythropoietin levels are low even with normal renal function, and as glycated hemoglobin levels increase, erythropoietin is further reduced [21]. SGLT2 inhibitors can inhibit energy expenditure in the microenvironment around the proximal tubule due to their effect of inhibiting ATP consumption by the Na+/K+ pump and restoring myofibroblasts to some degree to erythropoietin-producing fibroblasts. As a result, through recovering erythropoietin, hematocrit is increased [19]. The present results are consistent with the fact that both improved and nonimproved groups show an increase in Hct, regardless of whether diabetes is improved.

We focused on the movement of eGFR during the observation period because the two factors involved in the improvement of type 2 DM in the present analysis are also closely related to renal protection.

The change in eGFR two years after starting treatment was dramatic in the hyperfiltration group, about half of which improved to a level below the defined range of hyperfiltration. We understand this improvement owing to the drug's action related to the glomerular feedback mechanism. On the other hand, in the nonhyperfiltration group, there was a transient decline in the acute phase of the disease. Still, the decline was age-related after that, suggesting that the drug's effect was little in patients without hyperfiltration or with a normal eGFR. In the nonhyperfiltration group, there was a transient decline in the acute phase, which we consider to be due to a mixture of the acute effect of SGLT2-I and the increase in eGFR in some nephrons. Throughout the subsequent observation period, eGFR declined modestly, and the improvement in renal glomerular hyperfiltration and subsequent maintenance of filtration capacity with SGLT2-I treatment was considerably consistent with large cohort studies in terms of acute and chronic effects [22, 23]. However, the annual rate of decline in eGFR tended to be somewhat faster in our group than in the canagliflozin group in CANVAS [24]. Our results also showed the fastest decline among the papers on SGLT2-I as far as we could know. We treated about 90% of the patients in our study with insulin or insulin secretagogues and, unlike prospective studies, most of them were treated not with SGLT2-I alone but with polypharmacy. We speculate that elderly patients with impaired renal function, especially diabetic patients, are overloaded with hyperfiltration volume per single nephron due to a decreased number of nephrons. eGFR changes may vary greatly depending on population background, which may explain the faster eGFR decline in our study compared to other studies in the nonhyperfiltration group.

In this context, KDIGO (Kidney Disease Improving Global Outcomes), an international nephrology society, recommends SGLT2-I as the first-line drug for managing patients with type 2 DM and CKD in the 2022 edition of its guidelines [25]. The eGFR threshold for treatment was lowered from “30 mL/min/1.73 m^2^ or higher” to “20 mL/min/1.73 m^2^ or higher,” focusing on nephroprotective effects. In addition, there are growing expectations for the cardioprotective effects of the drug, such as correcting fluid overload by using SGLT2-I through osmotic diuresis and reducing cardiac afterload [26]. SGLT2-I is an invaluable diuretic that does not lose sodium against loop diuretics, and then we can use it even in patients with severe heart failure.

Our results in the present study are consistent with those of a large cohort study regarding renal protection [22, 23]. Still, in terms of improving glucose metabolism from the viewpoint of HbA1c, our results make us aware of the limitations of current pharmacotherapy for type 2 DM. Although the adverse effects of hyperinsulinemia have already been reported in the medical literature, insulin secretagogues and insulin continue to be actively administered as drug therapy for type 2 DM in clinical practice. Treatment with insulin and insulin secretagogues does not solve the problems in treating type 2 DM.

Hyperinsulinemia increases cardiovascular disease and mortality, cancer, advanced renal disease, and chronic inflammatory diseases [27, 28]. Hyperinsulinemia secondary to insulin resistance is an independent risk factor for vasospastic angina and obstructive coronary artery disease and is closely associated with early atherosclerotic lesions [29–31].

It is persuasive that compensatory hyperinsulinemia due to increased insulin resistance causes vascular injury in intervention studies with diabetic rats [32].

A 15-year cohort study reported that hyperinsulinemia and hyperglycemia increase cardiovascular and cancer mortality.

In this report, in addition to a stronger association with cardiovascular mortality, cancer mortality was significantly higher in diabetic patients than in the cohort as a whole. It is noteworthy that hyperinsulinemia is closely associated with hyperglycemia, highlighting the potential cancer-inducing effects of hyperinsulinemia. Taking the risks of hyperinsulinemia into consideration, we are just aware of the need for insulin-independent therapies.

Several research articles recommend combining SGLT2-I and BG preparations [33–35]. However, 95 patients in this study received SGLT2-I and insulin or insulin secretagogue simultaneously, while only five received SGLT2-I and BG preparations. Numerous recent reports demonstrate that antidiabetic polypharmacy does not reach sufficient improvement by insulin-independent therapy alone. In this study, the average decrease in HbA1c six months after treatment was 0.91% at best. The average HbA1c level was 8.19%, failing to achieve the treatment goal for HbA1c proposed by the Japan Diabetes Society.

We should set glycemic control goals for diabetic patients individually, considering age, duration of diabetes, health status, risk of hypoglycemia, and support system.

Davies et al. reported that hypoglycemia due to strict glycemic control also increases mortality and induces plaque instability and vascular dysfunction [36].

Therefore, it is the reality that the therapeutic target for HbA1c with tight glycemic control must at most be set at 7-8% or higher to avoid the risk of hypoglycemia, especially in older adults.

These findings recall the difficulties of strict hypoglycemic therapy, insulin-dependent pharmacotherapy limitations, and hyperinsulinemia's detrimental effects.

Therefore, SGLT2-I should be intensified alone or in combination with metformin to achieve insulin-independent antidiabetic pharmacotherapy that does not induce hyperinsulinemia.

Insulin-independent therapy should be the trump card to escape the chaos of type 2 DM treatment. Metformin is recommended as an adjunctive agent because it inhibits glycogenesis and protein synthesis by increasing AMP and activating protein kinases. It exerts its action through anticancer effects and modulation of the insulin pathway [37].

SGLT2-I may also provide a safe and effective antidiabetic treatment in combination with a mild carbohydrate-restricted diet.

Although there are some difficulties in continuing a carbohydrate-restricted diet, the combination of SGLT2-I and a mild carbohydrate-restricted diet could make continuous treatment possible.

SGLT2-I is an excellent antidiabetic agent that does not cause hyperinsulinemia. Since hyperinsulinemia is a high-risk factor for systemic organ damage and failure due to glucose toxicity, we must aim at achieving the treatment for organ protection by avoiding hyperglycemia and hyperinsulinemia.

### 4.3. Limitations

When using SGLT2-I, we recommend avoiding concomitant insulin or insulin secretagogues using metformin or insulin-independent antidiabetic agents and, in addition, using a low-carbohydrate diet. Unfortunately, our data did not allow us to present information on low-carbohydrate diets. Furthermore, this paper is limited to a literature review on low-carbohydrate diets. Since our study had an observation period of 2 years, we may need a more extended observation period to clarify the discussion of nephroprotective effects.

## 5. Conclusions

SGLT2-I significantly reduced HbA1c by approximately 1% six months after administration, followed by a slight increase in HbA1c. In general, SGLT2-I treatment maintained a leveling effect until two years after administration. Our results indicate that SGLT2-I is an excellent diuretic and antidiabetic drug, and we can use it safely, paying attention to dehydration, and other adverse events. In addition, SGLT2-I has already been reported to improve prognosis, and we expect SGLT2-I to reduce cardiovascular events and protect the kidneys by lowering blood pressure. Unfortunately, current doses alone are not potent enough to accomplish these goals. We must explore insulin-independent polypharmacy to power up treatment without dependence on insulin or insulin secretagogues. However, most insulin-dependent antidiabetic drugs induce hyperinsulinemia and increase insulin resistance and glucotoxicity. On the other hand, SGLT2-I is a new antidiabetic drug that can reduce hyperglycemia independent of insulin, which is entirely different from previous antidiabetic drugs. SGLT2-I has the potential to be a breakthrough in treating type 2 DM. However, achieving treatment with SGLT2-I alone takes pains. Establishing combined treatment of antidiabetic drugs that do not induce hyperinsulinemia, such as SGLT2-I, and a low-carbohydrate diet that raises less insulin is a crucial matter.

## Figures and Tables

**Table 1:** Background of target patients at starting treatment.

Patients (*n* = 236)※		
※ Patients have been taking SGLT2 inhibitors alone or with the other antidiabetic drugs for at least two years		
HbA1c (glico hemoglobin A1c)	9.1 ± 1.7	%
^*∗*^Sex (M/F)	152/84	
^*∗*^Age	61.4 ± 12.4	years
Male	61.6 ± 12.9	years
Female	61.0 ± 11.4	years
^*∗*^BMI (body mass index)	27.1 ± 5.2	kg/m^2^
Male	26.6 ± 5.1	kg/m^2^
Female	28.0 ± 5.2	kg/m^2^
^*∗*^Hct (hematocrit)	43.0 ± 4.2	%
TP (total protein)	7.50 ± 0.48	g/dL
ALB (albumin)	4.19 ± 0.40	g/dL
AST (aspartate aminotransferase)	27.4 ± 17.2	U/L
^*∗*^ALT (alanine aminotransferase)	33.1 ± 23.8	U/L
^*∗*^ *γ*-GTP (*γ*-glutamyl transpeptidase)	49.7 ± 51.4	U/L
^*∗*^TG (triglyceride)	186.8 ± 119.0	mg/dL
^*∗*^HDL-C (HDL-cholesterol)	51.6 ± 16.3	mg/dL
^*∗*^LDL-C (LDL-cholesterol)	118.1 ± 35.3	mg/dL
BUN (urea nitrogen)	16.62 ± 5.48	mg/dL
^*∗*^Cre (creatinine)	0.766 ± 0.255	mg/dL
^*∗*^BUN/Cre (BUN Cre ratio)	23.0 ± 7.0	
eGFR (estimated glomerular filtration rate)	81.0 ± 27.8	mL/min/1.73 m^2^
^*∗*^UA (uric acid)	5.63 ± 1.47	mg/dL
^*∗*^Na (sodium)	139.5 ± 2.6	mmol/L
^*∗*^K (potassium)	4.30 ± 0.41	mmol/L
	Mean ± SD	

^*∗*^Marks are items used as explanatory variables in logistic regression analysis.

## Data Availability

The data set used to support the results of this study is available upon reasonable request from the responsible author, Toshio Shimada (tshimada1946@yahoo.co.jp).
